# Supplementary material for: Estimating the risk and spatial spread of measles in populations with high MMR uptake: Using school-household networks to understand the 2013 to 2014 outbreak in the Netherlands
Source: PLoS Med. 2024 Oct 8;21(10):e1004466. doi: 10.1371/journal.pmed.1004466 (PMC11495615; doi:10.1371/journal.pmed.1004466)
Supplement: S1 Text — (DOCX) [file pmed.1004466.s001.docx]

**Supplementary information:** *Estimating the risk and spatial spread of measles outbreaks in populations with high MMR uptake: using school-household networks to understand the 2013 - 2014 epidemic in the Netherlands.*

*Evaluation of the resolution parameter*

We created partitions using the Leiden algorithm varying the resolution parameter between 0.1 and 1.0. We tested the partitions using three metrics:

Internal edge density: This metric measures the proportion of possible edges that are present in community C, expressed as:


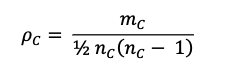


where [
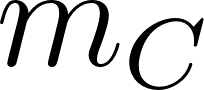
](https://www.codecogs.com/eqnedit.php?latex=m_%7BC%7D#0) is the number of edges internal to community [
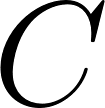
](https://www.codecogs.com/eqnedit.php?latex=C#0) and [
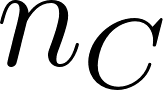
](https://www.codecogs.com/eqnedit.php?latex=n_C#0) is the number of

schools in community C. This takes a value between 0 and 1 and provides a quantification

of the absolute connectivity within the community, irrespective of connectivity with other

communities.

Modularity density: Modularity density normalises the quality function by the number of schools in the community, hence removing dependence on community size. This provides a better comparison of modularity between partitions with different community sizes. This is expressed as:


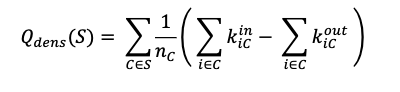


where [
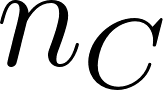
](https://www.codecogs.com/eqnedit.php?latex=n_C#0) is the number of schools in [
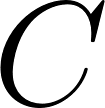
](https://www.codecogs.com/eqnedit.php?latex=C#0), [
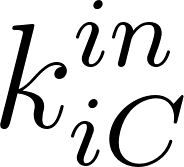
](https://www.codecogs.com/eqnedit.php?latex=k%5E%7Bin%7D_%7BiC%7D#0) is the degree of node i within [
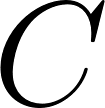
](https://www.codecogs.com/eqnedit.php?latex=C#0) (edges to schools inside the community) and [
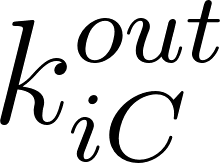
](https://www.codecogs.com/eqnedit.php?latex=k%5E%7Bout%7D_%7BiC%7D#0). is the degree of node i outside [
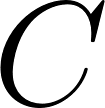
](https://www.codecogs.com/eqnedit.php?latex=C#0) (edges to schools outside the community) for a partition [
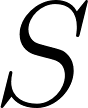
](https://www.codecogs.com/eqnedit.php?latex=S#0).

Newman Girvan Modularity: A classic metric of how strongly defined communities in a partition are from the rest of the graph. This is calculated as:


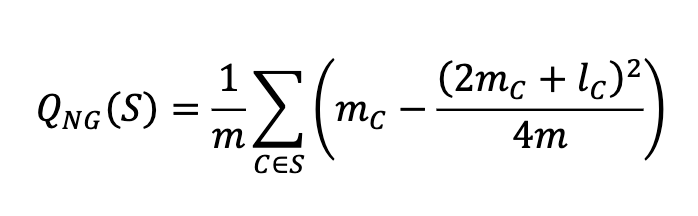


where [
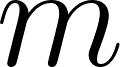
](https://www.codecogs.com/eqnedit.php?latex=m#0) is the number of graph edges, [
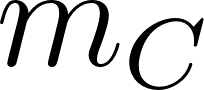
](https://www.codecogs.com/eqnedit.php?latex=m_C#0) is the number of community’s edges, [
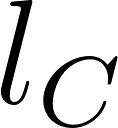
](https://www.codecogs.com/eqnedit.php?latex=l_C#0) is the number of edges from schools in [
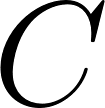
](https://www.codecogs.com/eqnedit.php?latex=C#0) to schools outside [
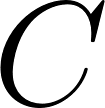
](https://www.codecogs.com/eqnedit.php?latex=C#0).

Surprise: Surprise is a quality metric assuming that edges between nodes emerge according to a hyper-geometric distribution. According to the Surprise metric, the higher the score, the less likely that the communities detected occurred at random and therefore the better the quality of the partition.

We found that all metrics were optimised when the resolution parameter was set to 1.0 (Figure S1), which results in the “unmodified” Leiden algorithm.

**
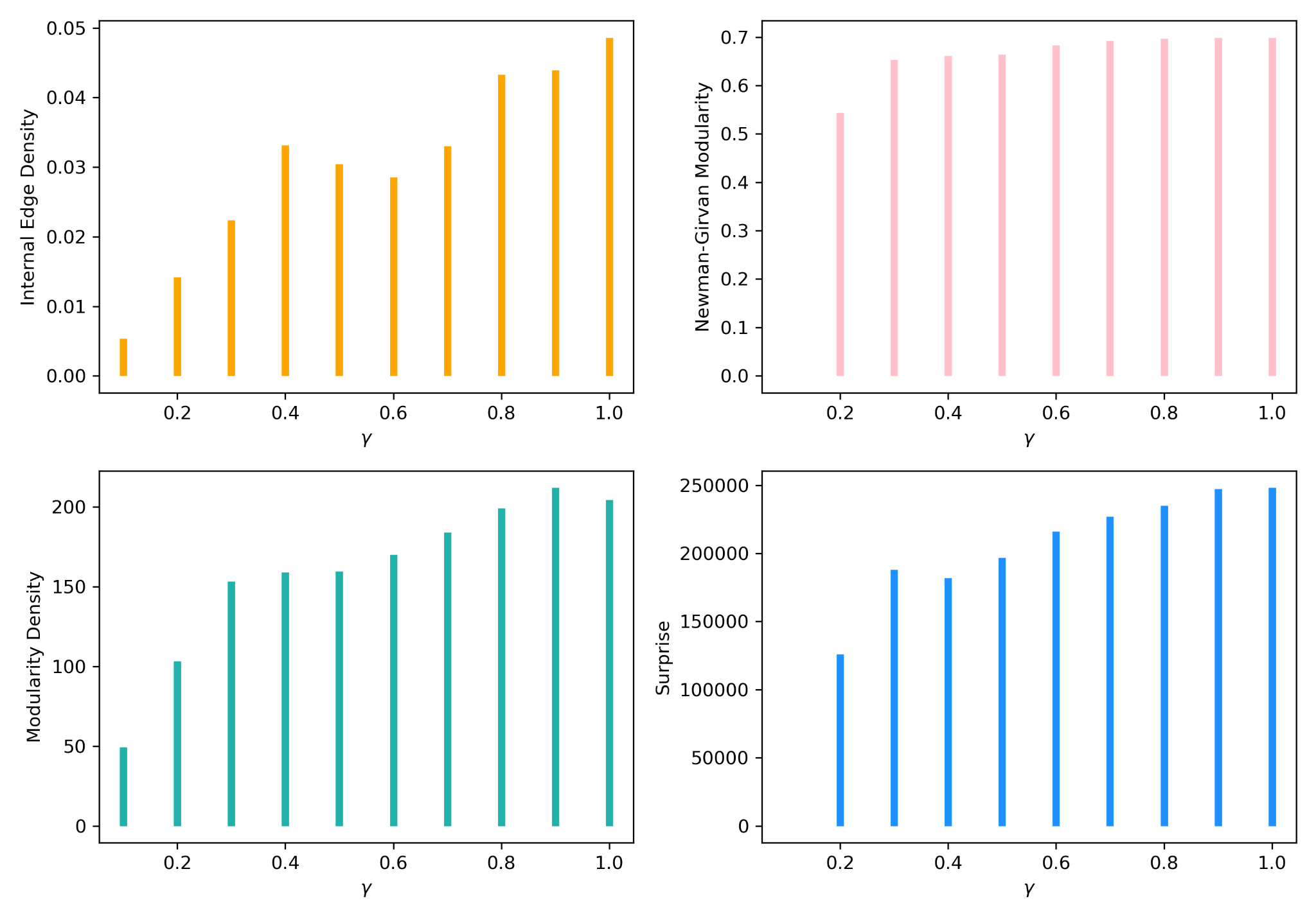
**

**Figure A.** Quality metrics for various values of resolution parameter for partitions recovered using the modified Leiden algorithm. Panels A to D show the scores for Internal Edge Density, Newman-Girvan Modularity, Modularity Density and Surprise respectively.

*Translation of contact network to a school based transmission model*

The basis of the model is that the network of contact between schools can provide an estimate of the probability that individual school outbreaks can seed an outbreak in each neighbouring school.

For each pair of neighbouring schools, we calculated the probability that an outbreak could be seeded in school $i$ given that an outbreak does occur in adjacent school $j$. First we consider the probability of transmission between siblings, in the event that one is infectious and the other is susceptible, to be a set value $q$. The probability that the child from school $j$ is infected is denoted by $P_{j}^{I}$ and the probability that the child from school i is susceptible by $P_{i}^{S}$. The probability that a single infected student in school i causes a large outbreak in that school is $P_{i}^{OB}.$ The probability of an outbreak in school $j$ leading to an outbreak in school $i$ through each unique contact pair that link schools $i$ and $j$ is:

$$P_{j}^{I}P_{i}^{S}qP_{i}^{OB}$$

The probability that the child in school $j$ is infected, $P_{j}^{I}$, was assumed to be equal to the proportion of the school children infected by the outbreak in that school. We assumed that this is defined by the solution of the final size equation34:

$R_{j}\left( \infty\right)=\left( 1-V_{j} \right){(1-e}^{-\left( 1-V_{j} \right)R_{0}R\left( \infty\right)}$)

Where $V_{j}$ is the vaccination coverage in school $j$.

The probability that the child in school i is susceptible is equal to the proportion of school i that remains unvaccinated, $(1-V_{i})$.

We took the probability of an outbreak occurring in that school as a result of this transmission to be equivalent to the final size equation for an outbreak, which assumes a Poisson distributed offspring distribution.

The probability that none of the unique contact pairs causes an outbreak in school i can be written:

$$\prod_{All pairs} \left( 1-P_{j}^{I}P_{i}^{S}qP_{i}^{OB} \right)=\left( 1-P_{j}^{I}P_{i}^{S}qP_{i}^{OB} \right)^{C_{ij}}$$

Therefore, the probability that at least one contact pair causes an outbreak in school i is the complement of this:

$$P_{trans, ij}=\left[ 1-\left( 1-P_{j}^{I}P_{i}^{S}qP_{i}^{OB} \right)^{C_{ij}} \right]$$

This provides a basis upon which to model simulations of outbreaks across networks of schools in different settings.

*Alternative model 2: Spatial interaction between schools*

The spatial interaction model was designed to have a broadly equivalent spatial distribution of contact between schools but with otherwise naïve interaction (i.e. no preference for contact within particular denominations or between tiers of education etc.).

We formulated interaction between schools as an exponentially distributed distance relation with an additional weighting on schools determined by their degree in the empirical school network. We selected the parameterisation of this distribution by visually matching the resultant distribution of the *distance between schools connected by contact pairs* over the entire network. The value of [
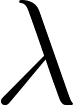
](https://www.codecogs.com/eqnedit.php?latex=%5Clambda#0) that best reflected the data-driven network was 0.65, which was aimed at matching the peak of the distribution of contact described in the data.


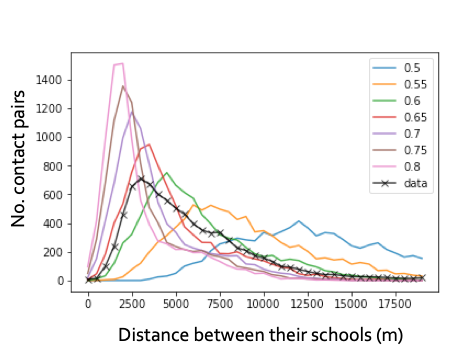


**Figure B** The distribution of distance between schools connected by contact pairs. The coloured lines show interaction based on spatial interaction models with parameters between 0.5 and 0.8. Black line with cross markers shows the distribution in the household links from the data.

*Sensitivity analyses*

To establish the sensitivity of outbreak size and distribution to the values of within school reproduction number (R_0_) and sibling to sibling transmission probability (q), we re-ran the analysis with R_0_ of 12 and 18 (15 in main analysis) to represent extremes of the estimated R_0_ range in literature. We also ran the analysis with a reduced value of q = 0.5 (0.9 in main analysis) to reflect the impact if recent studies of household secondary attack rates resulted in over estimates.

First, it is possible to quantify the impact of changing R_0_ final size of school-level outbreak (and risk of outbreak in a new school per introduction) by simply solving the final size equation for each proportion vaccinated and each value of R_0_ (Figure S4). It is clear that the variability is very low in all except low susceptibility populations.


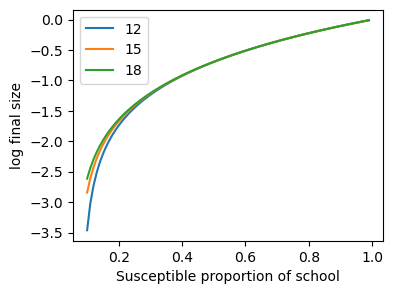


**Figure C** Log of estimated final size of within-school outbreaks depending on reproduction number and proportion of school susceptible.

When we re-ran the full simulation analysis for the different values of R_0_ we found the the final size of the outbreak initiated at the school in which cases were first detected in 2013 varied between 23,989 (23,711-24,416 IQR) for R_0_ of 12 and 24,526 (24,252-24,897) for an R_0_ of 18. For an q value of 0.5 the final size was 23,497 (23,340-24,267IQR). This compared to the value in the main analysis of 24,310 (24,052 – 24,736 IQR).

When we consider spatial distribution of infections we found that the wROC values for the baseline model (data driven network model and school level uptake) varied between 0.94 sensitivity and 0.91 specificity for R_0_ of 12 and 0.94 sensitivity and 0.91 specificity for R_0_ value of 18. When we set q to 0.5 we found 0.94 sensitivity and 0.91 specificity. These compare to 0.93 sensitivity and 0.92 specificity in the main analysis.

Figures S4 to S6 show the spatial distribution for the sensitivity analyses. Overall the values vary by a small degree and the conclusions are unaffected.


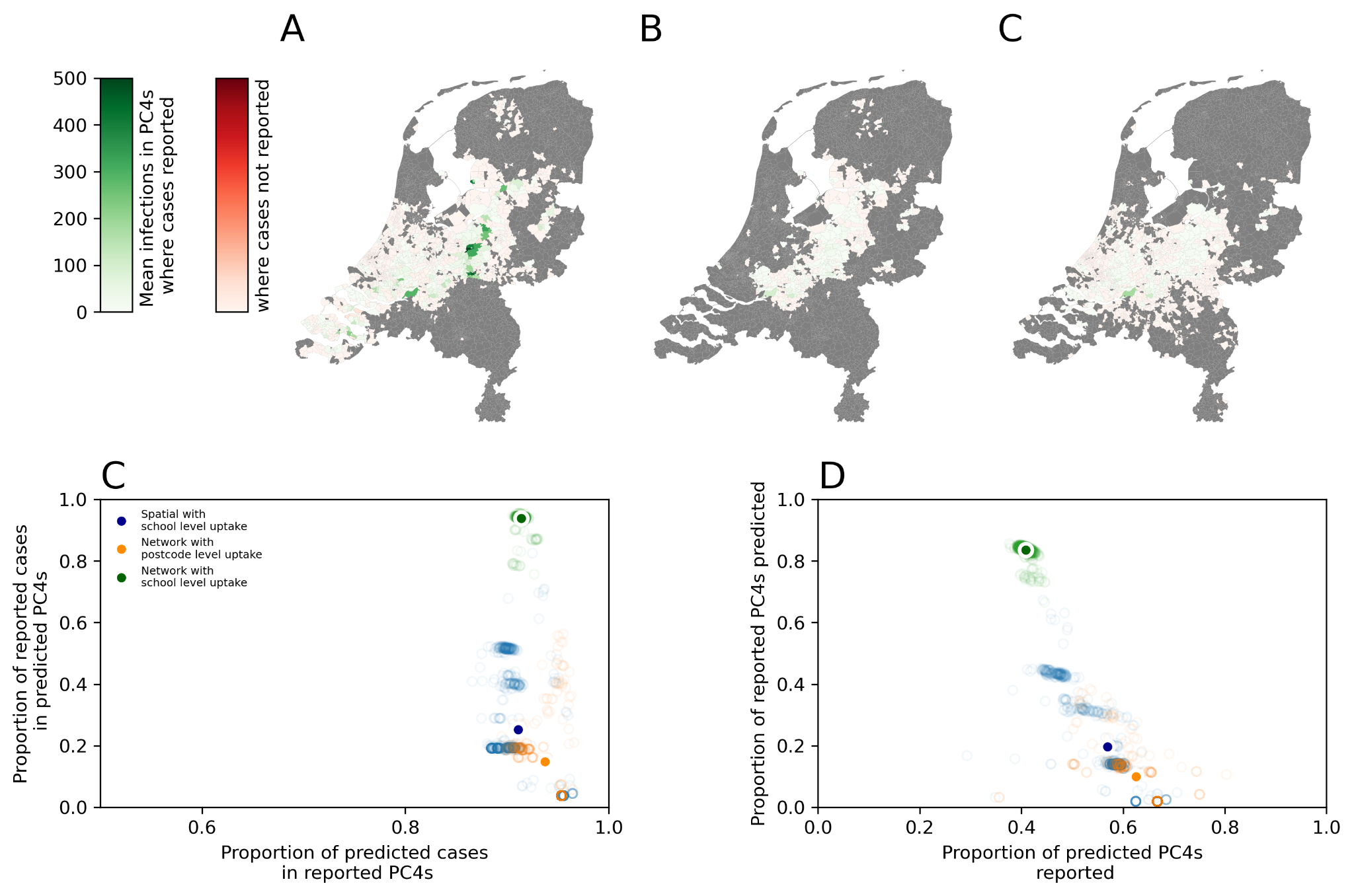


Figure D: Sensitivity study results with *R_0_*=12. Mean number of cases across 1000 simulated in each PC4 region with a reporting rate of 10% (from estimates in literature). A) The baseline model: School data network with school level uptake, B) Alternative model 1: School data network with PC4 level uptake, C) Alternative model 2: Spatial network with school level uptake, D) weighted sensitivity and specificity, and E) unweighted sensitivity and specificity of the baseline and alternative network models.


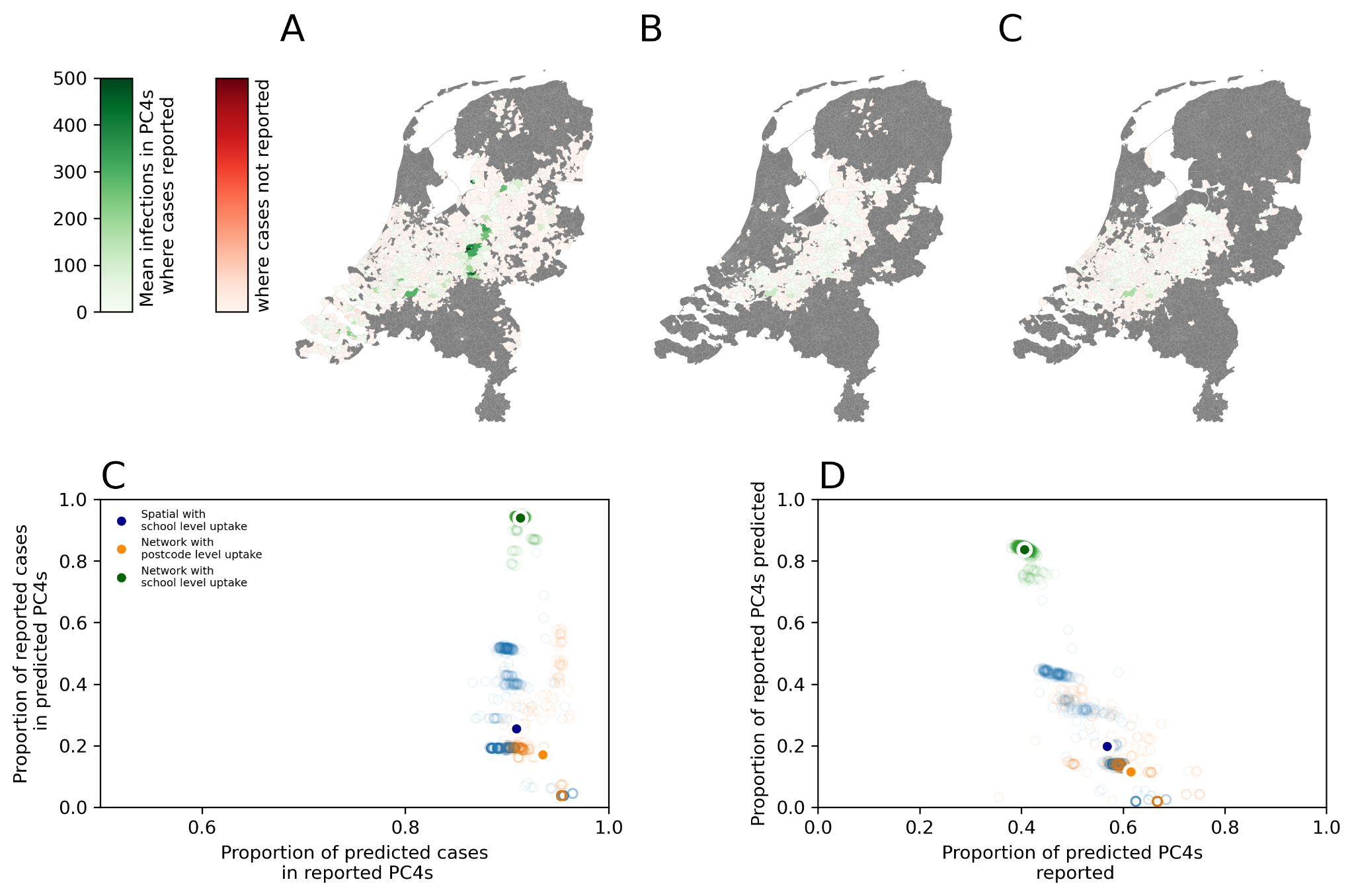


Figure E: Sensitivity study results with *R_0_*=18. Mean number of cases across 1000 simulated in each PC4 region with a reporting rate of 10% (from estimates in literature). A) The baseline model: School data network with school level uptake, B) Alternative model 1: School data network with PC4 level uptake, C) Alternative model 2: Spatial network with school level uptake, D) weighted sensitivity and specificity, and E) unweighted sensitivity and specificity of the baseline and alternative network models.


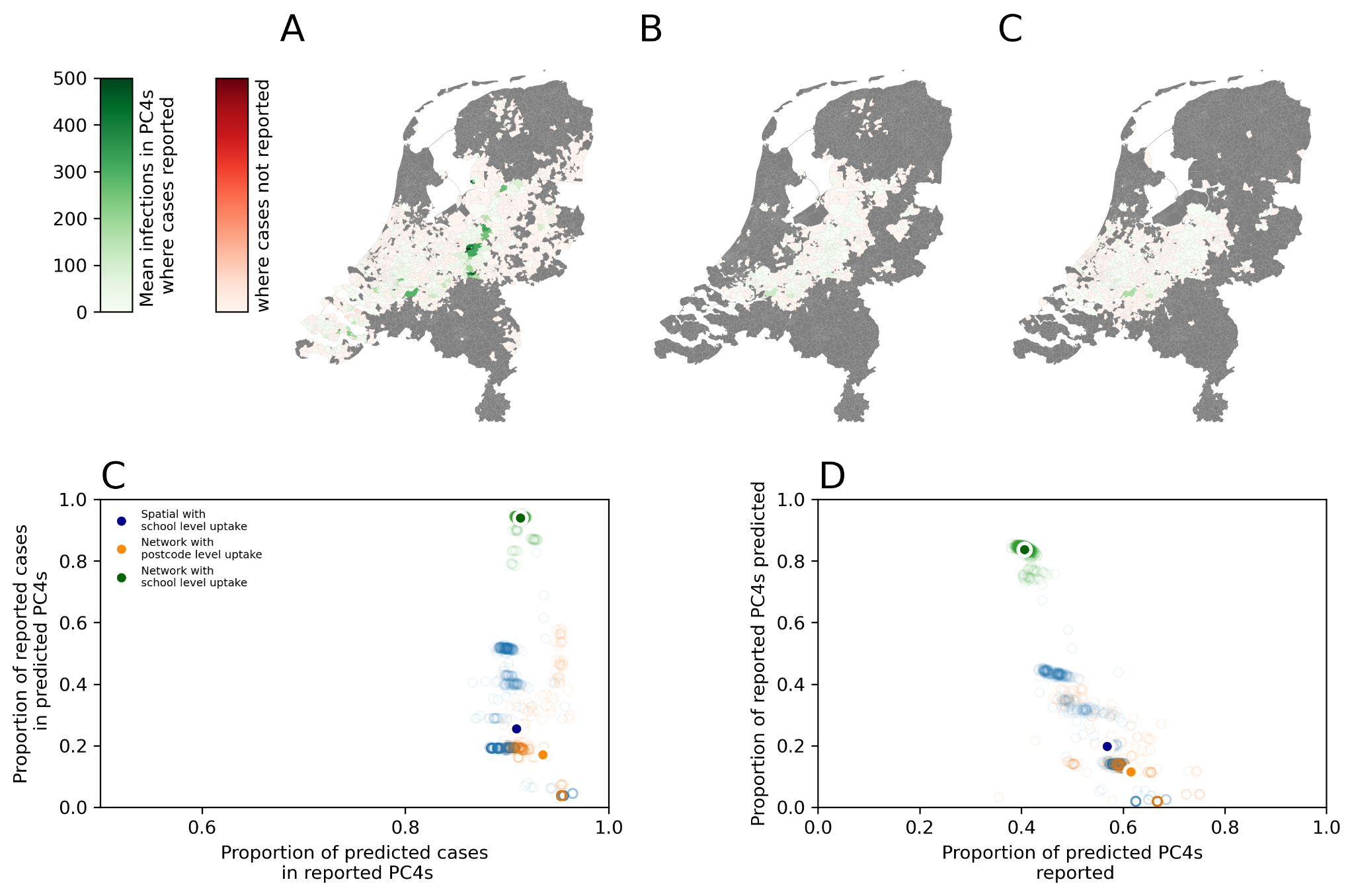


Figure F: Sensitivity study results with *q*=0.5. Mean number of cases across 1000 simulated in each PC4 region with a reporting rate of 10% (from estimates in literature). A) The baseline model: School data network with school level uptake, B) Alternative model 1: School data network with PC4 level uptake, C) Alternative model 2: Spatial network with school level uptake, D) weighted sensitivity and specificity, and E) unweighted sensitivity and specificity of the baseline and alternative network models.

*Supplementary figures and tables*


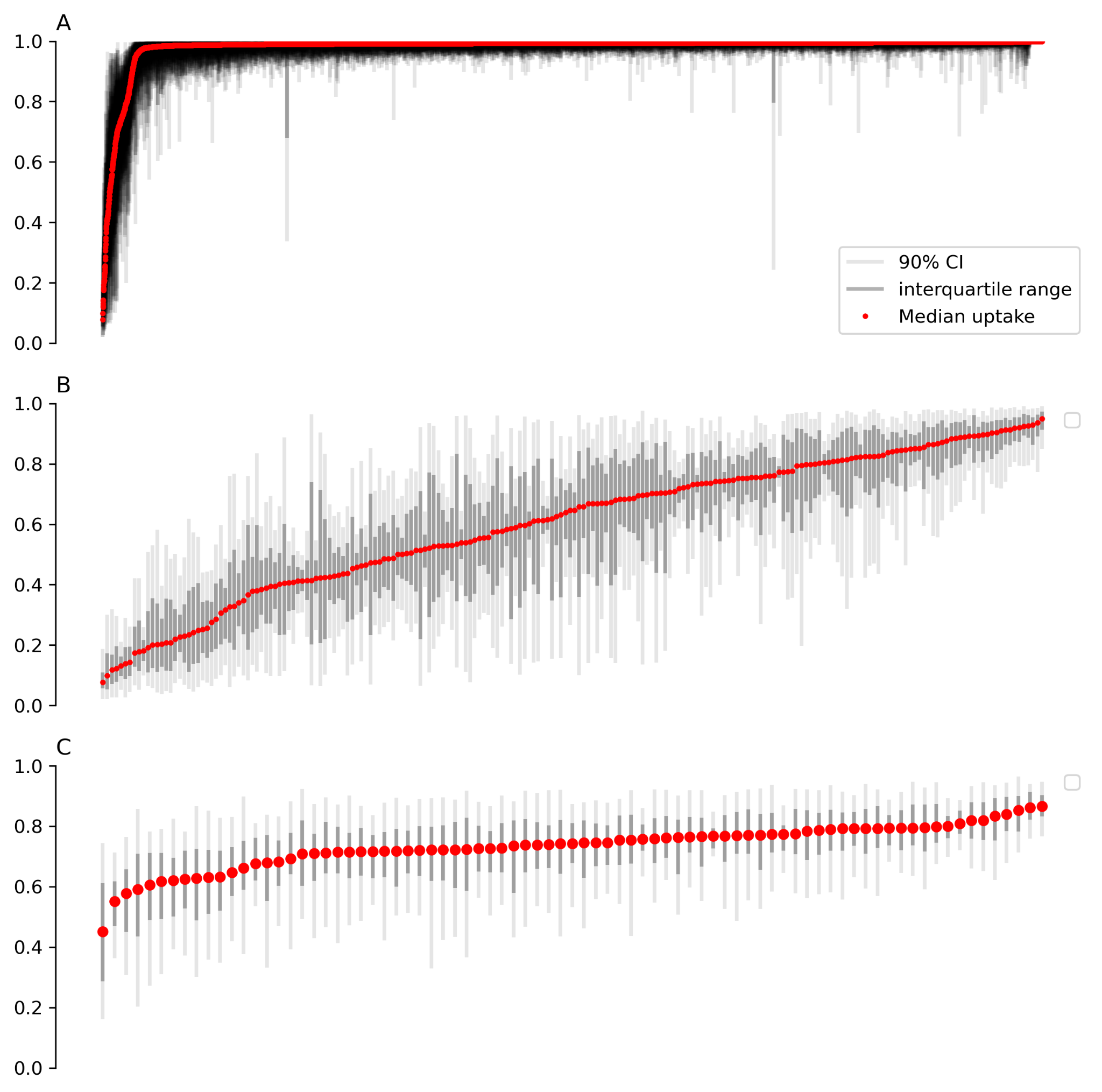


**Figure G** Estimates of vaccine uptake by school, showing median values and interquartile range for each school estimated by Klinkenberg et al. (2021) for A) all schools, B) orthodox Protestant schools and C) Anthroposophic schools.


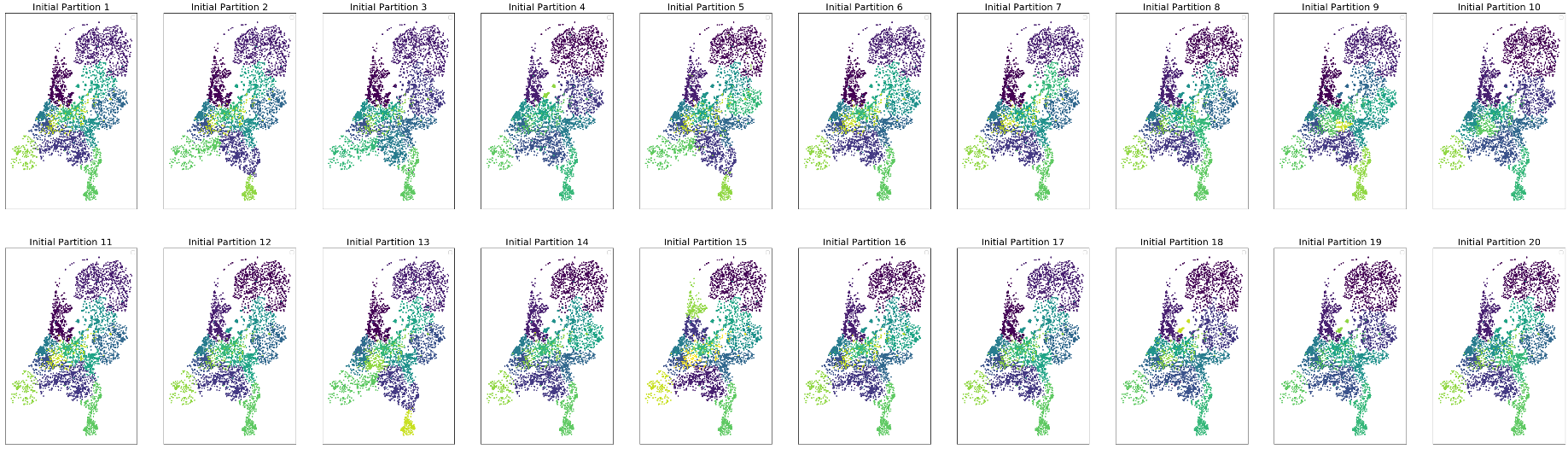


Figure H The 20 initial partitions each panel shows a partition as the locations of schools in the Netherlands with a colour indicating the community of schools in the partition.


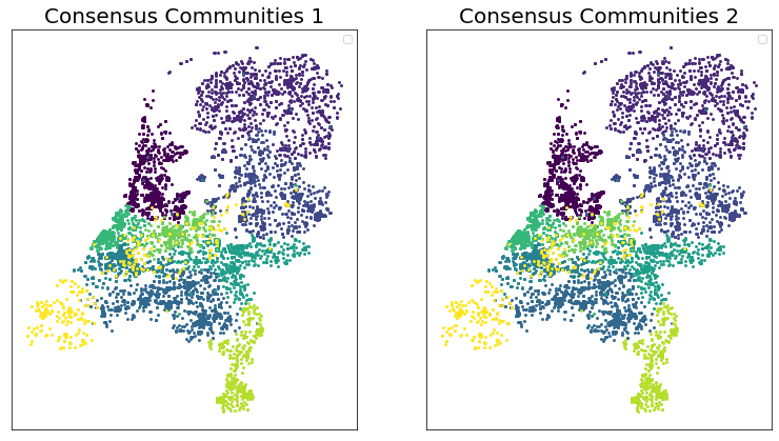


Figure I The partitions from the first round of the ensembling algorithm, each panel shows a partition as the locations of schools in the Netherlands with a colour indicating the community of schools in the partition.


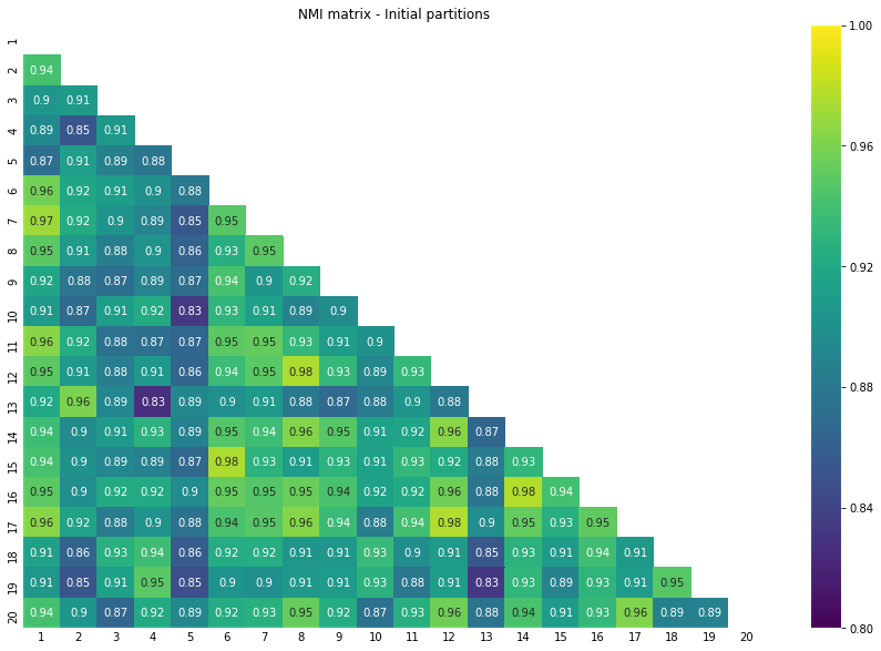


Figure J A matrix normalised mutual information (NMI) between the initial partitions.


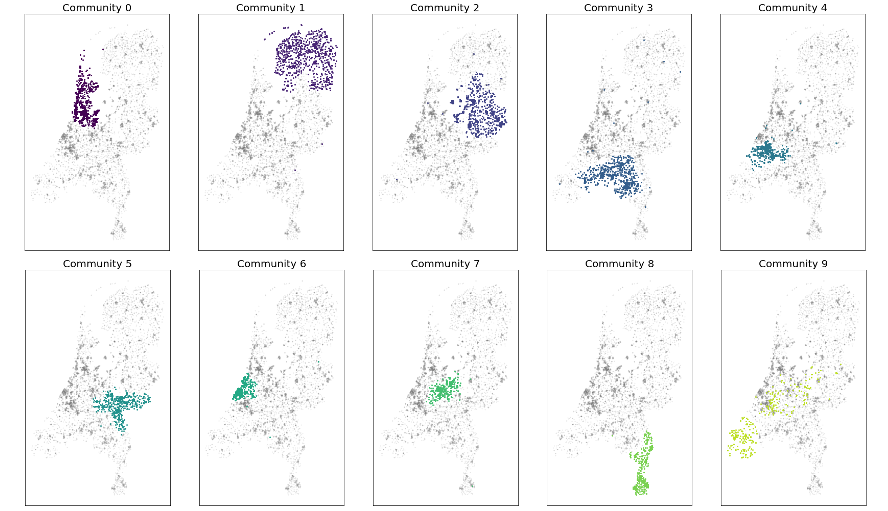


Figure K. final consensus partition, each panel shows a schools in each community in the consensus partition as coloured points, grey points show the locations of other schools.

Table A school denominations

| School identity | *Dutch name* | *Schools* | *Primary* | *Secondary* |
| --- | --- | --- | --- | --- |
| Public school | Openbaar | 2810 | 2466 | 344 |
| Roman Catholic | Rooms-Katholiek | 2554 | 2258 | 296 |
| Protestant | Protestants-Christelijk | 2147 | 1904 | 243 |
| Special educational philosophy | Algemeen bijzonder | 807 | 579 | 228 |
| Orthodox Protestant | Reformatorisch | 208 | 181 | 27 |
| Reformed liberated | Gereformeerd vrijgemaakt | 118 | 118 | 0 |
| Anthroposophic | Antroposofisch | 81 | 70 | 11 |
| Islamic | Islamitisch | 44 | 43 | 1 |
| Interconfessional | Interconfessioneel | 21 | 15 | 6 |
| Reformed Liberated | Gereformeerd | 18 | 0 | 18 |
| Evangelical | Evangelisch | 16 | 12 | 4 |
| Hindu | Hindoeistisch | 6 | 6 | 0 |
| Other | Overige | 4 | 0 | 4 |
| Jewish | Joods | 2 | 2 | 0 |
| Moravian Church | Evangelische Broedergemeenscha | 2 | 2 | 0 |
| Potestiant/Evangelical | Protestants-Christelijk/Evange | 1 | 0 | 1 |
| Jewish Orthodox | Joods orthodox | 1 | 0 | 1 |
| Potestiant/Reformed | Protestants-Christelijk/Reform | 1 | 0 | 1 |
