# Supplementary material for: Estimating the risk and spatial spread of measles in populations with high MMR uptake: Using school-household networks to understand the 2013 to 2014 outbreak in the Netherlands
Source: PLoS Med. 2024 Oct 8;21(10):e1004466. doi: 10.1371/journal.pmed.1004466 (PMC11495615; doi:10.1371/journal.pmed.1004466)
Supplement: Attachment — Submitted filename: PLoSReview_Munday180324.pdf [file pmed.1004466.s003.pdf]

Anticipating the risk and spatial spread of measles in populations with high MMR uptake: Using school-household networks to understand the 2013 - 2014
Review

The authors propose that large measles outbreaks may occur in countries with very high vaccine coverage due to clustering of people who reject vaccination. The mechanism of clustering is an association between belief systems and negative attitudes toward vaccination, which lead to schools of certain denominations being strongly linked through households, thus creating a larger than expected, connected, unvaccinated population.

These hypotheses are investigated with a combination of data analyses to build and investigate a network, and simulations of outbreaks on the empirical and alternative networks. They find support for their proposed mechanism, and the methodology for their study is interesting and innovative. I think this paper represents a worthy contribution to the field. The quality of the data on which it is based is not so easy to come by, which sadly limits the potential for exploiting these ideas in other settings. However, the pathway for disease spread is believable and needs to be considered in other epidemic contexts.

To my knowledge, the authors are aware of the most relavant literature. Studies of similar situations in the context of the US may, however, enrich their discussion and open routes of contrast for future work [1-4], and a study for the Belgian situation also presents relevant similarities [7].

Their data analyses show a significant association between households and denominations of schools attended by their children, specially for the dominant religions. According to their contrast between contact and physical distance, this link brings closer together (few contacts away) schools of the same denomination that may be geographically distant.

This was a fun and interesting read. The general idea of the paper is sound and original. Subject to a few corrections and clarifications, I think this piece of work is well worth publishing.

In my opinion, some assumptions need to be further justified or modified:

1. The probability with which a student in school j may be infectious is taken to be the final proportion of infected individuals. This seems somewhat high for a probability that refers to a particular moment of the epidemic (and not to the probability of being infectious over the course of the whole epidemic as reflected by final size estimates). Moreover, larger schools will have outbreaks that last longer, and this is in no way captured by taking merely final size estimates. Indeed, it might be a good idea to consider how a large school may continue to seed infection in other schools once an outbreak starts there.

2. The final size equation is said to be $R_j(\infty) = (1 - V_j)e^{-(1-V_j)R_0R(\infty)}$ where the fraction of susceptibles present in each school at the start of the epidemic is equated to the unvaccinated fraction (in school j, $S_j(0) = (1 - V_j)$). However, the expression provided in the manuscript is that for the final susceptible fraction $S_j(\infty) = 1 - R_j(\infty)$. Also, as far as I am aware $S_j(0)$ does not appear in the argument of the exponential function for the final size (see for example p. 22 of [5]). Maybe the authors are discounting the R_0 by vaccination in order to plug in the effective reproductive number, in which case it is worth mentioning and justifying this in the text. It worries me that the expression for the final size provided in the paper may indeed reflect the value used in simulations, because I expect it would alter their outcome in some important manner.

Index j is missing from $R(\infty)$ (should be $R_j(\infty)$).

3. The probability of a newly infected individual in susceptible school i causing an outbreak is defined as $p_i^{OB} = (1 - 1/((1 - V_j)R_0)$. This means that it has a value below the herd immunity threshold $S_c = 1 - 1/R_0$. Thus, the critical fraction of susceptibles needed to observe an outbreak is even lower than it would generally be for measles. It makes sense to estimate the susceptible fraction needed to observe an outbreak and incorporate it into this probability, but it is unclear to me how the current definition of p_i^{OB} is doing that. (Maybe my confusion stems from the fact that I do not fully understand how simulations are done.)

I do not understand the sentence starting in line 208. If $p_i^{OB} = p_i^I$, then it is defined as the final size of the epidemic in that school, which seems an even greater overestimatation for p_i^{OB} than in the case mentioned earlier (p_j^I). Moreover, this information contradicts what is said in page 4 of the supplementary information, so it may be just some confusion.

4. Related to this issue, I find the value for R_0 somewhat high for the years under consideration. It may be worthwhile to argue why it is assumed to be 15 and what the effect is of giving it other values. Similarly, a value of $q = 0.5$ is taken as the probability of infection passing from one sibling to another, but there is no explanation for this choice. It might be better to select estimates for household transmission (or for a similar kind of setting) from the literature.

5. While understanding the relevance of taking vaccination levels into account, other aspects of the proposed model parameterisation seem too specific. Exploring a somewhat wider range of parameter values for the proposed network would provide more certainty. The only parameter that I am aware is being changed are vaccination coverage estimates, but I am not convinced that this is producing a wide enough exploration (within reasonable limits) of parameter space. Since there is actual uncertainty as to what is the final size of an epidemic in a given school, there is room for selecting that value from a distribution...

6. Draw a comparison of vaccine uptake between the baseline and alt 1 models so that it can be better understood how different they turn out to be.

I find some of the methodological details insufficient and/or unclear:

1. Please provide a step by step algorithm explaining the way in which a simulation is set up and run. I understand that the authors refer to their recent paper to fill in some of these details, but the method is sufficiently new to warrant repeating the explanation here.

2. Please give enough details on how alternative models 1 and 2 are built, so that readers are able to reproduce them. For example, what defines the direction of edges in figure S3?

Minor points:

I understand that the authors are leaving references to figures for the results section, but they might be forthcoming in other parts of the text: line 187, 261 and in other places. I suggest referring in the methods section to the figure or table where the results of your analyses are shown.

Provide references that support your assertion/detail your source in lines: 20, 25, 30, 56.

The sentence starting "To create..." in line 243 is unclear.

Table 1 Might be a good idea to list the different religions in English or leave them always in Dutch in the text (depending on the policy followed by the journal). Also, I don't think it's necessary to give the translation in each instance where a denomination is mentioned.

lines 371, 375 I suggest sticking to the model monickers used this far.

line 399 I understood the data being used covered somewhere around 2/3 of the school children population (according to what is said in earlier in the text).

line 405 Total incidence was in the right order of magnitude.

line 454 What percentages are being given? Is that the fraction of that age group affected by measles? Something else?

Typos:

line 61 'vaccination' (singular) or 'vaccines'.

line 62 clustering *of*

line 65 'given way' (no hyphen)

line 67 Maybe introduce a period and start a new sentence after reference [13]

line 98 *this allows us to* better understand...

line 195 'R'ecovered.

line 317 Capitalise 'Orthodox' after period; Reformatorisch has an extra e.

line 393 I believe 'some' should be 'same'

line 401 I suggest 'tight' instead of 'close' line 403 Change word order: 'both sensitivity and specificity of'

line 459 'since the contribution...' what? (Seems like the thought was left incomplete, or maybe you meant to say 'given the contribution...'?)

Name the main network used in the paper (maybe household-school link network)

References:

[1] Aloe C, Kulldorff M, Bloom BR. Geospatial analysis of nonmedical vaccine exemptions and pertussis outbreaks in the United States. Proc Natl Acad Sci U S A. 2017 Jul 3;114(27):7101-7105. doi: 10.1073/pnas.1700240114. Epub 2017 Jun 20. PMID: 28634290; PMCID: PMC5502604.

[2] Feikin DR, Lezotte DC, Hamman RF, Salmon DA, Chen RT, Hoffman RE. Individual and community risks of measles and pertussis associated with personal exemptions to immunization. JAMA. 2000 Dec 27;284(24):3145-50. doi: 10.1001/jama.284.24.3145. PMID: 11135778.

[3] Gangarosa EJ, Galazka AM, Wolfe CR, Phillips LM, Miller E, Chen RT, Gangarosa RE. Impact of anti-vaccine movements on pertussis control: the untold story. The Lancet. 1998 Jan 31;351(9099):356-61.

[4] Salmon DA, Haber M, Gangarosa EJ, Phillips L, Smith NJ, Chen RT. Health consequences of religious and philosophical exemptions from immunization laws: individual and societal risk of measles. Jama. 1999 Jul 7;282(1):47-53.

[5] Keeling MJ, Rohani P. Modeling infectious diseases in humans and animals. Princeton university press; 2011 Sep 19.

[6] Rodgers DV, Gindler JS, Atkinson WL, Markowitz LE. High attack rates and case fatality during a measles outbreak in groups with religious exemption to vaccination. The Pediatric infectious disease journal. 1993 Apr 1;12(4):288-91.

[7] Kuylen E, Willem L, Broeckhove J, Beutels P, Hens N. Clustering of susceptible individuals within households can drive measles outbreaks: an individual-based model exploration. Scientific reports. 2020 Nov 12;10(1):19645.
