# Supplementary material for: Estimating the risk and spatial spread of measles in populations with high MMR uptake: Using school-household networks to understand the 2013 to 2014 outbreak in the Netherlands
Source: PLoS Med. 2024 Oct 8;21(10):e1004466. doi: 10.1371/journal.pmed.1004466 (PMC11495615; doi:10.1371/journal.pmed.1004466)
Supplement: Attachment — Submitted filename: Response to reviewers.pdf [file pmed.1004466.s004.pdf]

Response to reviewers' comments for: **Estimating the risk and spatial spread of measles in populations with high MMR uptake: using school-household networks to understand the 2013 - 2014 outbreak in the Netherlands.**

Thank you for giving us the opportunity to revise our manuscript. We have made every effort to address each of the points raised and are pleased with how this has improved the paper. We are excited to hear how you and the reviewers receive these changes. Below are detailed responses to each of the comments and requested revisions.

Comments of the editorial board:

The manuscript sparked a lively debate in terms of how the study is currently presented, particularly with regard to the focus on religious affiliation of the included schools. The consensus among the editorial team was that there is potentially too much emphasis placed on the religious affiliations and that this might detract/distract from the main point of the study regarding the impact of unvaccinated children (in a more general sense) on outbreaks.

We were also concerned that the presentation might have the potential to offend readers by suggesting that people who subscribe to a certain religion are more likely to be vaccine hesitant (eg, line 328: "…Orthodox Protestant (Reformatorisch) and Anthroposophic (Antroposofisch) … the two faith identities with populations who systematically refuse vaccination."

Thank you for highlighting this important issue. We agree that it is essential we keep the manuscript focussed on the impact of clustering among unvaccinated children and don't offend readers. However, to frame this issue correctly, we would like to draw the attention of the editorial board to the fact that in numerous previous publications about low vaccine uptake in the Netherlands the Orthodox Protestant and Anthroposophical communities are explicitly named and discussed. In that sense our study is not an isolated case. It is well established that, particularly in the case of the Orthodox Protestant group, the lower vaccine uptake is deeply related to the beliefs, teaching and politics of the group itself. Having said that, we have amended our manuscript in several ways. On reflection, we felt it necessary to highlight that membership of these groups was not the only characteristic that was associated with lower MMR uptake in the Netherlands. Furthermore, we rephrased the description of the groups such that there is more emphasis on the association between group membership and vaccine refusal, as opposed to a generalised relationship. As indeed both groups contain vaccinated children, and we want to affirm that vaccination acceptance is an individual decision regardless of social group. Besides this, we have sharpened the description of the groups - highlighting that the Orthodox Protestant groups represent a religious community whereas the Anthroposophic group is a much looser definition community related to a set of views and philosophies. Although less explicit, we also improved the introduction of the differentiated school system in the Netherlands, with a better introduction of the situation that two third of the schools in the Netherlands have a particular identity. Subsequently we have adapted this throughout the manuscript, which we hope will focus the attention of the reader on the role and position schools with a Orthodox Protestant or Anthroposophic identity have in the national school-household network, and therefore keep the narrative focussed on the clustering.

We have also removed the line you mention explicitly in your comment. The text now reads:

“which are the two school identities with relatively low vaccine uptake compared to the general Dutch population ”

Please feel free to contact me directly at kjanin@plos.org <mailto:kjanin@plos.org> if you like to discuss the above further.

Thank you for our pleasant discussion.

In line with the Academic editor, we also feel it important to review the languages used around specific denominations and be mindful not make overgeneralized statements, as noted above.

We also felt it would be helpful if you could provide more context of the general Dutch school system (in the Introduction) for the benefit of our international readership.

Thank you for drawing our attention to this. We have now added a paragraph in the introduction describing the dutch school system in more detail and added some more information at the beginning of the method section. We hope these additions provide sufficient information for the international reader to understand the Dutch school system and our manuscript.

In the introduction:
“The Netherlands has a differentiated school system, which allows parents to organise and/or choose primary and secondary schools freely, often in line with their household values or faith. Approximately a third of state-funded primary and secondary schools are organised by the government and do not promote any specific religious or ideological stance (the identity referred to as “Openbaar”). The remaining two thirds of state-funded schools are affiliated with one of 27 distinct identities linked to a faith or (educational) philosophy. “

In the method section:
“Children in the Netherlands enter primary school at the age of 4, where they remain for 8 years. At the age of 12 they enter secondary school, which is usually separate from primary. Secondary school is divided into three academic tiers: VMBO (Pre-vocational training), HAVO (General senior education) and VWO (Pre-university). Students remain studying in these contexts until they are 16, 17 and 18 years old respectively. “

For the model, we feel that the temporal dynamics of the outbreak are important (per the comments of Reviewer #1) and need to be carefully considered in your revision

Thank you for your comment. We agree that the text was unclear and understand the confusion. To clarify: the model itself does not take into account a temporal component that is scalable to the progression of a real (ongoing) outbreak, because we do not evaluate the temporal dynamics of the outbreak, but rather the overall risk posed by the end of the outbreak by evaluating final size and geographic spread. Therefore the framework operates such that each generation of schools is infected in discrete steps. In the discussion we refer to the timing of outbreaks, not in reference to the progression of a single outbreak but rather the interval between outbreaks. We simply mean to suggest that with updated school level immunity

estimates in schools and projections of how this might change, the framework could be used to evaluate the risk of outbreaks in the years/decades after the previous outbreak. We have adapted the discussion, we have removed the reference to outbreak timing in the headline paragraph and we have combined reference to repeated analysis in future years to evaluate risk, placing the single paragraph immediately before the discussion of limitations. It reads:

“Further, since the variation in outbreak size is due to structural differences in the population, it is likely that future outbreaks in these communities would follow similar patterns, if the structure of the school system remains comparable in years to come. More detailed exploration of these dynamics could be studied by considering the evolution of immunity in schools. For example, our analysis could be repeated on an annual basis utilising the data held in the Dutch vaccination registry and considering the additional immunising effect of previous outbreaks in combination with administrative data of the department of education. Such analysis could assist national and local public health teams in their assessment of risk and subsequently which groups of parents/children to target for national and local campaigns to reduce this risk.“

Table 1: Dutch labels are used for Denomination descriptions. Please provide descriptors in English for the benefit of our international readership (feel free to also keep the Dutch labels)

Thank you for highlighting this. We have added english translations for each of the denominations (now identity) in the table.

Please capitalise 'Orthodox' when describing 'Orthodox Protestant" religion (as part of the proper name). Please check and amend.

Thank you for clarifying this point. We have now amended the manuscript accordingly.

TITLE:
Please substitute the word ‘Anticipating’ with ‘Estimating' or something similar (given this is a modelling study).

Thank you for the suggestion, we have now amended the title.

ABSTRACT:
Please structure your abstract using the PLOS Medicine headings: Background, Methods and Findings, Conclusions. Please remove all other subheaders.

Thank you for bringing this to our attention, we have now amended the abstract to reflect this format.

Abstract Background:
Provide the context of why the study is important. The final sentence should clearly state the study question.

Abstract Methods and Findings:
Please include the study design
In the last sentence of the Abstract Methods and Findings section, please describe the main limitation(s) of the study's methodology.

Thank you, this was added.

AUTHORS SUMMARY
At this stage, we ask that you include a short, non-technical Author Summary of your research to make findings accessible to a wide audience that includes both scientists and non-scientists. The Author Summary should immediately follow the Abstract in your revised manuscript. This text is subject to editorial change and should be distinct from the scientific abstract. Please see our author guidelines for more information: https://journals.plos.org/plosmedicine/s/revising-your-manuscript#loc-author-summary<https://journals.plos.org/plosmedicine/s/revising-your-manuscript#loc-author-summary>
Ideally each sub-heading should contain 2-3 single sentence, concise bullet points containing the most salient points from your study.

Thank you for inviting us to contribute this element. We have included it in the manuscript after the abstract.

In the final bullet point of 'What Do These Findings Mean?' Please include the main limitations of the study in non-technical language.

Thank you, this was added.

ACKNOWLEDGMENTS/ DECLARATIONS
Please remove all statements apart from acknowledgements, author contributions and abbreviations from the end of the main manuscript and include these only in the relevant parts of the manuscript submission form. Funding, competing interest, and data availability will be compiled as metadata.

Thank you for clarifying this. We have now removed the surplus text.

STUDY DESIGN:
Of all authors who submit a modelling study we ask for inclusion of specific items, derived from Geoffrey P Garnett, Simon Cousens, Timothy B Hallett, Richard Steketee, Neff Walker. Mathematical models in the evaluation of health programmes. (2011) Lancet DOI:10.1016/S0140-6736(10)61505-X.

Please ensure all the items listed below are included with your manuscript. Please review the list below and confirm/revise as necessary:

i) Please provide a diagram that shows the model structure, including how the disease natural history is represented, the process and determinants of disease acquisition, and how the putative intervention could affect the system.

In the previous version we included a figure to detail the assembly of the contact network between schools in the manuscript, and a diagram of the outbreak simulation process itself in the supplementary material. To avoid doubt we have enhanced the figure of the outbreak simulations and included it in the main manuscript. We also included a small panel to summarise the 'natural history' of infection between schools.

ii) Please provide a complete list of model parameters, including clear and precise descriptions of each parameter, together with the values or ranges for each, with justification or the primary source cited, and important caveats about the use of these values noted.

We have added a table with the model parameters with sources for these values cited.

iii) Please provide a clear statement about how the model was fitted to the data [including goodness-of-fit measure, the numerical algorithm used, which parameter varied, constraints imposed on parameter values, and starting conditions].

We do not fit this model to data, but rather parameterise the data entirely from detailed schools data and transmission values from literature. We clearly explain these elements. We then evaluate the data against epidemiological data.

iv) For uncertainty analyses, please state the sources of uncertainties quantified and not quantified [can include parameter, data, and model structure].

We extensively expound the limitations of our model choices in the discussion.

v) Please provide sensitivity analyses to identify which parameter values are most important in the model. Uncertainty estimates seek to derive a range of credible results on the basis of an exploration of the range of reasonable parameter values. The choice of method should be presented and justified.

To address this comment we have rerun the model several times to show the impact of the parameter choices on the core components of the transmission model. We show that the variance introduced by the uncertainty in the data-driven vaccine-uptake parameterisation has the greatest impact on the risk of transmission between schools.

vi) Please discuss the scientific rationale for this choice of model structure and identify points where this choice could influence conclusions drawn. Please also describe the strength of the scientific basis underlying the key model assumptions.

We believe that the main narrative of the paper deals with this subject directly, as our analysis is focussed on the comparison between alternative models.

DATA AVAILABILIY:

We note your write: “The measles case data is available on request: datastewards@rivm.nl<mailto:datastewards@rivm.nl>. … Measles case data is not publicly available but is available on request from RIVM. Requests should be made to osiris.aiz@rivm.nl <mailto:osiris.aiz@rivm.nl>.” Please clarify where researcher can gain access to the measles case data.

Thanks for raising this, we included the first address by error as we were initially unsure of the correct specific address. We will amend the resubmission.

For data that are not freely available, please describe briefly the ethical, legal, or contractual restriction that prevents you from sharing it.

Thanks for raising this, we have added this in the text.

As a guide: PLOS Medicine requires that the de-identified data underlying the specific results in a published article be made available, without restrictions on access, in a public repository or as Supporting Information at the time of article publication, provided it is legal and ethical to do so.
"The Data Availability Statement (DAS) requires revision. For each data source used in your study:
a) If the data are freely or publicly available, note this and state the location of the data: within the paper, in Supporting Information files, or in a public repository (include the DOI or accession number).
b) If the data are owned by a third party but freely available upon request, please note this and state the owner of the data set and contact information for data requests (web or email address). Note that a study author cannot be the contact person for the data.
c) If the data are not freely available, please describe briefly the ethical, legal, or contractual restriction that prevents you from sharing it. Please also include an appropriate contact (web or email address) for inquiries (again, this cannot be a study author).

Academic Editor

Thank you for taking the time to review our manuscript. We very much appreciate your insight and have endeavoured to improve the paper in line with your comments.

The methods will need to be made more accessible. (Please note that we require methods to be describe in sufficient detail for another researcher to replicate the study)

Thank you for your comment. We have expanded the methods to include a more detailed description of the model in the main text. We have also corrected some typos pointed out by the reviewers, which we understand improves clarity a great deal. Other than the calculation of edge weights in the various networks we construct, which we describe in detail, the methods used are well established in the field of network analysis.

Wasn't clear to if the models allowed for mixing between primary and secondary schools, e.g. households with siblings in primary and secondary schools (I would assume so, but could be more clearly stated).

Thank you for your comment. Yes the model includes interaction between primary and secondary schools. We have added the following sentence to the end of the paragraph where we discuss the contact network to make this explicit.

'The resulting network describes the number of unique contact pairs between each pair of schools in the Netherlands, including between primary and secondary schools.'

ROC estimates for sensitivity and specificity from models need to report uncertainty.

Thank you for raising this, we have now added the 95% credible intervals from the simulated outbreaks to the text.

In the Discussion it is also important that the authors reflect appropriately on generalisability outside the Netherlands. (mention of England only, but should consider how this methodology could be applied in a wider range of settings, and what adaptations might be needed).

Thank you for your comment, in line with this and the reviewers comments we have extended the section in the discussion to elucidate what further adaptations would be required to carry out similar work in other settings.

Overall, it is important to ensure that the authors are mindful to not blame specific denominations for "causing" outbreaks, despite their low vaccine uptake and importance to transmission identified in models. This can be done by being careful in wording (e.g. "... schools seeded outbreaks..." etc

Thank you for raising this. We are deeply aware that vaccine practices correlated with particular identities is a sensitive issue. We have discussed this at length with the editor. We understand that although these practices are important for Public Health - they are not an essential part of the narrative in this paper and discussion of these risks being interpreted as blame. We have revised the manuscript extensively to remove any reference to vaccine hesitancy or vaccine refusal and to ensure there is no implication of blame when we simulate outbreaks initiated in certain schools. In particular, relating to your comment we have revised the section about outbreak size by initial school such that we no longer refer to outbreaks seeded 'by' schools but rather seeded 'in' schools. We believe this change reduces the risk that this will be misinterpreted as blame.

"The overall risk posed by an outbreak seeded in each particular school was quantified by finding the distribution of final outbreak size. For both the school data and spatial networks, the majority of schools had a very low mean outbreak size as no sustainable transmission was observed in any simulation.
For the full network model, the maximum mean outbreak size was 158 schools and 24,324 children and was seeded in with a school with the Orthodox Protestant identity(Figure 5). In general, outbreaks seeded in schools with this identity were larger. particularly where the seed schools had very low vaccination coverage. Outbreaks seeded in schools with an Anthroposophic identity generally remained much smaller, with a maximum of 5 schools and 598 children.

Outbreaks simulated on alternative model 2, with a spatially derived network, were generally much smaller. In comparison to the full network model, there remained a general trend with vaccine uptake, however, in many cases some outbreaks seeded schools with very low uptake were much smaller. The largest outbreaks using alternative model 2 had a mean of 25 infected schools and 6,645 children infected. In this simulation the largest outbreaks were seeded in a school with the Orthodox Protestant identity . However, the difference between outbreaks seeded in the schools with the Orthodox Protestant and Anthroposophic identities were much less substantial than for the full network model, with a number of outbreaks seeded in Anthroposophic identity schools becoming larger than that predicted by the model derived from the school data. The largest outbreak seeded in a school with Anthroposophic identity included 23 schools and 5,762 children. Notably outbreaks seeded in certain Anthroposophic identity schools were comparable to those seeded in Orthodox Protestant identity schools with similar vaccine uptake.
"

Figure 2 will need a bit of work to improve legibility and interpretation

Thank you for your comment. We have now broken this figure up into separate figures for each panel and placed them in the supplementary information

Reviewer #1:

Reviewer #1: Thank you for the invitation to review the manuscript by Munday et al. Given school-specific MMR vaccine uptake, the authors created a national school-household network to quantify the outbreak risk of measles in the Netherlands. By simulating measles outbreaks on the network parameterized on 2013 data, the authors were able to explain the observed size and spatial distribution of 2013/14 outbreak, and identified that the highly connected orthodox Protestant schools associated with low vaccine uptake contribute to large outbreaks. The findings are important for understanding why measles outbreaks still occur in countries with high MMR vaccine uptake, and for archiving the WHO goals of eliminating measles.

Thank you for taking the time to review our manuscript. We are deeply grateful for your suggestions and comments.

However, I am concerned about whether it is adequate for reproducing the historical outbreak by comparing only the final size and spatial distribution without considering the epidemic curve. The authors acknowledge in the limitations that the current model cannot model temporal dynamics, but why can the results have important implications for understanding the timing of large measles outbreaks in the Netherlands (Lines 421-422)?

Thank you for your comment. We have addressed your question in our response to the editorial board.

The authors think that their analysis can be repeated on an annual basis to assess the outbreak risk (Line 410). However, in the following discussion on implications, the authors also believe that the future outbreaks are likely to follow similar patterns among communities, if the population structure remains comparable in years to come (Lines 442-444). I am a bit confused whether it is necessary for the public health authorities to conduct the analysis annually.

Thank you for raising this. We realise that the text is confusing. As discussed in the previous comment: Although we expect vaccine attitudes have remained broadly similar, the changes in immunity profile in schools due to of vaccine derived and naturally acquired immunity combined with the network of contact between schools, we anticipate that the dynamics of potential outbreaks is likely to change over time. We have added the paragraph referenced in the previous comment to clarify this point.

Moreover, the manuscript is full of formatting typos, including capitalization, punctuation, abbreviations, references etc., which the authors should carefully proofread. Below are those typos to be fixed.

Thank you for your detailed review of the text. We are tremendously grateful for the effort you have gone to to improve the text. We have implemented your suggestions in almost all of the cases. In cases where our solution differed from your initial suggestion or requires more explanation we have added a specific comment below

Line 339, I cannot find the "scatter plots of network distance against geographic distance". Please insert a reference to the figure.
Thank you for raising this to our attention. This short paragraph actually refers to an old version of the figure, we removed these plots since they show the same information as the distance ratios. We have revised the text to remove the reference to this old figure.

Lines 345-347, how 5.18×10-3 and 4.81×10-3 are compared with 0.54 pairs-1km-1 and 3.83 pairs-1km-1 as they have different units and magnitudes?

Thank you for raising this typographical error, a previous draft reported in m as opposed to km and this was not updated. It is now amended in the text.

Line 460, move "tend to be smaller" between "settings" and ",".

Thank you for raising this. I disagree with this one. I think it needs to read that because they tend to be smaller they are unlikely to form key links on the network. I have added another comma instead.

Line 501, MMR is only the abbreviation of Measles, Mumps and Rubella.

Thank you for your comment, we disagree, MMR is frequently used to refer to the vaccine itself across the literature. However, your comment has raised the important point that we have been inconsistent in this use. We have now amended this so that MMR refers to the vaccine explicitly throughout the text.

In main text, a gaussian offspring distribution is assumed; however, here a Poisson distribution is assumed. Which one is correct?

Thank you for raising this, the offspring distribution is assumed to be poisson by virtue of the approximations we make, this was a typo in the supplement, we have now corrected.

Reviewer #2

Thank you for taking the time to review our manuscript, we very much appreciate your comments and have enjoyed responding. We hope you find that our solutions and responses satisfy your queries.

The authors propose that large measles outbreaks may occur in countries with very high vaccine coverage due to clustering of people who reject vaccination. The mechanism of clustering is an association between belief systems and negative attitudes toward vaccination, which lead to schools of certain denominations being strongly linked through households, thus creating a larger than expected, connected, unvaccinated population.

These hypotheses are investigated with a combination of data analyses to build and investigate a network, and simulations of outbreaks on the empirical and alternative networks. They find support for their proposed mechanism, and the methodology for their study is interesting and innovative. I think this paper represents a worthy contribution to the field. The quality of the data on which it is based is not so easy to come by, which sadly limits the potential for exploiting these ideas in other settings. However, the pathway for disease spread is believable and needs to be considered in other epidemic contexts.

To my knowledge, the authors are aware of the most relavant literature. Studies of similar situations in the context of the US may, however, enrich their discussion and open routes of contrast for future work [1-4], and a study for the Belgian situation also presents relevant similarities [7].

Thank you for bringing these studies to our attention. We agree with your suggestion that we could explicitly refer to comparable vaccine uptake patterns elsewhere. We now refer to the [1-4] in the introduction:

'*This increase in unprotected children raises fears of increased measles outbreak risk as has been documented in the past for measles and other highly infectious pathogens [9–12]'*

And in the discussion:

'*This method could also be applied in other settings where vaccine uptake is strongly related to particular social groups [14], for example in the United States, where non-medical vaccine-mandate exemption also has the potential to generate clusters of unvaccinated children [9–12]. However, further application of this framework relies on detailed school data being made available in the relevant settings.*'

Their data analyses show a significant association between households and denominations of schools attended by their chil- dren, specially for the dominant religions. According to their contrast between contact and physical distance, this link brings closer together (few contacts away) schools of the same denomination that may be geographically distant.

This was a fun and interesting read. The general idea of the paper is sound and original. Subject to a few corrections and clarifications, I think this piece of work is well worth publishing.

In my opinion, some assumptions need to be further justified or modified:

1. The probability with which a student in school j may be infectious is taken to be the final proportion of infected individuals. This seems somewhat high for a probability that refers to a particular moment of the epidemic (and not to the probability of being infectious over the course of the whole epidemic as reflected by final size estimates). Moreover, larger schools will have outbreaks that last longer, and this is in no way captured by taking merely final size estimates. Indeed, it might be a good idea to consider how a large school may continue to seed infection in other schools once an outbreak starts there.

Thank you for your comment. You are absolutely right that the model should consider the overall risk of transmission between schools. This is what we do by calculating the risk of transmission from any one infected in an outbreak in school i to a child in school j who lives in the same household. We do not model this process in time, but rather take a 'generational' approach such that the risk is calculated once based on the final size of the outbreak in school i. This means that the probability does represent the risk of transmission over the course of the entire epidemic (as you suggest would be the correct course) rather than at a particular point in the epidemic. We have adapted the text to make this clearer.

from:
Subsequently an infected school, j, can infect connected school, i, with probability $P_{trans,\ ij}$

to:
Subsequently, over the course of the school outbreak in school j, a new outbreak can be initiated in connected school i, with probability $P_{trans,\ ij}$

2. The final size equation is said to be $R_j(\infty) = (1 - V_j)e^{-(1-V_j)R_0 R(\infty)}$ where the fraction of susceptibles present in each school at the start of the epidemic is equated to the unvaccinated fraction (in school j, $S_j(0) = (1-V_j)$). However, the expression provided in the manuscript is that for the final susceptible fraction $S_j(\infty) = 1 - R_j(\infty)$. Also, as far as I am aware $S_j(0)$ does not appear in the argument of the exponential function for the final size (see for example p. 22 of [5]). Maybe the authors are discounting the R0 by vaccination in order to plug in the effective reproductive number, in which case it is worth mentioning and justifying this in the text. It worries me that the expression for the final size provided in the paper may indeed reflect the

value used in simulations, because I expect it would alter their outcome in some important manner.

Thank you for raising this. We believe your concern is due to an inconsistency between the main text and the supplement. To alleviate your concern we will describe in detail the motivation for this expression. The final size of an outbreak in a completely susceptible population can be approximated by solving

$Rj(\infty) = 1 - e{-}R0\ R(\infty)$

Regarding the susceptible population. Under the assumption that immunity is well distributed in the population this approximation holds for partial immunity. The uncorrected expression:

$Sj(\infty) = e{-}R0\ R(\infty)$

will give the remaining susceptible fraction.

As you suggest we modify the expression with the effective reproduction number ((1-Vj)R0), so the expression now gives the fraction of the original susceptible population that remain after the outbreak. Hence the complement of this gives the fraction of susceptibles infected (similar to the final size equation above)

$1\text{-}e{-}(1{-}Vj\ )R0\ R(\infty)$

By multiplying by the proportion of the population initially susceptible we arrive at the proportion of the total population infected by the outbreak

$Rj(\infty) = (1{-}Vj\ )(1\text{-}e\text{^}{-}(1{-}Vj\ )R0\ R(\infty))$

This is what we include in the manuscript. We have, as you suggest, included reference to the effective reproduction number in the manuscript to clarify the relevance of (1-Vj)R0 to the reader.

Index j is missing from $R(\infty)$ (should be $Rj\ (\infty)$).

Thank you for highlighting this inconsistency - we have now amended.

3. The probability of a newly infected individual in susceptible school i causing an outbreak is defined as $pOB = (1 - 1/((1 - Vj\ )R0\ )$. This means that it has a value below the herd immunity threshold $Sc = 1 - 1/R0$. Thus, the critical fraction of susceptibles needed to observe an outbreak is even lower than it would generally be for measles. It makes sense to estimate the

susceptible fraction needed to observe an outbreak and incorporate it into this probability, but it is unclear to me how the current definition of pOB is doing that. (Maybe my confusion stems from the fact that I do not fully understand how simulations are i done.) I do not understand the sentence starting in line 208. If pOB = pI , then it is defined as the final size of the epidemic in that school, which seems an even greater overestimatation for pOB than in the case mentioned earlier (pI). Moreover, this information ij contradicts what is said in page 4 of the supplementary information, so it may be just some confusion.

Thank you for your comment. Yes there was a discrepancy in the manuscript. We had initially implemented that the probability of an outbreak was 1 - 1/Reff) which is the correct approximation for an outbreak with a geometric offspring distribution. However, we changed this to be in line with our approximation for the final outbreak size - which assumes a poisson distributed offspring distribution.

Following Diekmann, Heesterbeek, Britton, *mathematical tools for understanding infectious disease dynamics,* section 3.3: In the case of a poisson distributed offspring distribution the probability of a 'minor' outbreak (i.e. not major) is approximated by z=exp(R0(1-z)) which is directly analogous to the final-susceptible-fraction equation.

We adapted this in the same way as our estimate of the final size equation to modify for partially vaccinated population (assuming 100% vaccine efficacy) to estimate the probability of a major outbreak, hence POB = PI. (Aside: you are right to suggest that the outbreak probability reduces to below that with no vaccination since the expected number of offspring reduces per infected individual).

4. Related to this issue, I find the value for R0 somewhat high for the years under consideration. It may be worthwhile to argue why it is assumed to be 15 and what the effect is of giving it other values. Similarly, a value of q = 0.5 is taken as the probability of infection passing from one sibling to another, but there is no explanation for this choice. It might be better to select estimates for household transmission (or for a similar kind of setting) from the literature.

Thank you for your comment. We understand your concern that we do not explore the parameter space of R0 and q.

Firstly R0. We use R0=15 as an estimate which lies within the frequently cited range of 12 to 18. R0 has an impact on the outbreak size and the probability of an outbreak. However, in the range of a very high R0, between 12 to 18, the impact is very limited and therefore does not have a substantial impact on results, hence since this study does not seek to model the dynamics of the pathogen in detail we do not see it necessary to complicate the results by including this range. To address the concerns, we have included a sensitivity analysis in the supplement to show that the variation in our key results is (indeed) very low and does not affect our conclusions.

Secondly, the value of q. Thank you for raising this, when we first developed this model there was limited evidence for what the secondary attack rate in households might be. We tested the model for different values of q and found that for a reasonable range (0.3-0.9) there was very little impact on the overall results. Prompted by the reviewer questions, we found a 2023 meta-analysis that suggested it to be as high as 90%. For this reason we have rerun the analysis with a q of 0.9 and incorporated these results in the manuscript. Note that the conclusions remain unchanged - demonstrating the robustness of our results to variation in household transmission. We include the results for q=0.5 in the supplement as a sensitivity study.

5. While understanding the relevance of taking vaccination levels into account, other aspects of the proposed model parameterisation seem too specific. Exploring a somewhat wider range of parameter values for the proposed network would provide more certainty. The only parameter that I am aware is being changed are vaccination coverage estimates, but I am not convinced that this is producing a wide enough exploration (within reasonable limits) of parameter space. Since there is actual uncertainty as to what is the final size of an epidemic in a given school, there is room for selecting that value from a distribution…

Thank you for your comment. This comment seems to connect the previous two comments. Regarding the suggestion to draw the final size from a distribution, we already in essence do this by drawing vaccine uptake from a distribution. The impact of the variation in vaccine uptake estimates by school is far more substantial than the impact of the possible variation between an R0 of 12 and 18, hence we suggest that keeping the fixed value of R0 is sufficient to describe the uncertainty in the context of this study - where we do not seek to model the detailed spread of measles but rather show the overall impact of clustering of unvaccinated children is schools combined with the clustering of these schools on the school-household network.

Reviewer #3

Thank you for taking the time to review our manuscript. We are grateful for your insights and have done our best to address them. Our detailed responses are below.

This is an interesting paper concerned with an important topic and the network analysis is useful on its own and well linked to the transmissibility element of this problem. I have some comments that require clarification and amendment.

Please add the evidence for the q=0.5 assumption. This seems like a crucial component, driving the transmission in the overlapping part.

Thank you for your comment, please see our response to reviewer 2 on the same topic.

The Poisson distribution for the within school contact rate (supplement end of page 4) is presumably a Poisson process like in typical epidemic models?

Thank you for your question. We refer to the contact distribution in the schools as poisson distributed since the approximations we use for the final size and probability of a major outbreak are consistent with an outbreak with a poisson distributed offspring distribution.

It would be useful to summarise in a table the parameters (for each of the 3 models) that are actually calibrated to fit the observed outbreak data.
If feasible this table may also include the network key characteristics, like the λ parameter mentioned in supplement page 7 (above fig S2).

We agree that although there are only two parameters a table would make them more accessible to the reader, thank you for the suggestion. We have now incorporated this in the manuscript.

Is the Ro=15 value assumed fixed with no uncertainty?

Thank you for your question. We have replied to a similar question by reviewer 2.

The Gaussian distribution is somewhat unusual for the offspring, have the authors tried distributions defined on the positive (and perhaps integer) numbers?

Thank you for raising our attention to this. The approximations relating to within-school transmission are consistent with a poisson distributed offspring distribution. We have corrected the text accordingly.

The authors do not refer to the large methodological/statistical/theoretical literature and the connections of their model to related models like Britton, Kypraios T. and O'Neill P.D. (2011), Statistical models for epidemic models with three levels of mixing and the series of papers on Network epidemic models with two levels of mixing by Frank Ball and Peter Neal

Thank you for your comment, we have now included reference to this paper in the discussion as well as other similar agent based models.

"Further, our framework could be used to provide parameterisation for more detailed models which explicitly model infection between individual hosts [26,48,49], or by applying methods to account for within school transmission dynamics using more parsimonious approaches [50]. "

Which of these (very detailed) data are publicly available?

Thanks for your comment, we share the school network data via the public github repository. The measles case data is not publicly available but can be requested from RIVM via the email address in the data availability statement.

It would be useful to clarify all the aspects (code+data) of the reproducibility of this work.

Thank you for your comment, all code is available on a public GutHub repository. Some data is available on the repo, the remaining data can be requested from RIVM as detailed in the Data and Code Availability section:

The code and school network data is available at https://github.com/jdmunday/SchoolsMeaslesNL. The measles case data is available on request: datastewards@rivm.nl. School population statistics are publicly available from:

https://www.duo.nl/open_onderwijsdata/index.jsp. Measles case data is not publicly available but is available on request from RIVM. Requests should be made to osiris.aiz@rivm.nl.
