# Supplementary material for: Estimating the risk and spatial spread of measles in populations with high MMR uptake: Using school-household networks to understand the 2013 to 2014 outbreak in the Netherlands
Source: PLoS Med. 2024 Oct 8;21(10):e1004466. doi: 10.1371/journal.pmed.1004466 (PMC11495615; doi:10.1371/journal.pmed.1004466)
Supplement: Attachment — Submitted filename: Response to editor and reviewers.pdf [file pmed.1004466.s006.pdf]

# Response to editor and reviewers

We are tremendously grateful to the editors and reviewers for their time and effort in reviewing our manuscript and believe it is now a much better paper. As requested, we have addressed the points raised in your email. We detail the changes we have made below:

EDITORIAL COMMENTS:

1. MAIN COMMENT: The editorial team concurs with reviewer 1, that some copy edit issues remain. Please have thorough copy edit of your manuscript. In places, 'emotive language' remains and we invite you to reduce it as much as possible to data driven and factual statements (see e.g. point 3).

Thank you for raising this. We have now copy edited our manuscript – particularly the introduction and discussion/conclusion sections. We have made a concerted effort to identify emotive language and reduce it to more objective equivelant statements – please refer to the tracked changes manuscript for all of these changes. In particular the most substantial changes (and in addition to the below points) we in the discussion as follows:

**Line 477:**
**From:**
*The increased connectedness between the Orthodox Protestant identity schools combined with low coverage of MMR creates large clusters of unvaccinated children, which would be expected to increase the risk of large outbreaks of Measles, Mumps and Rubella amongst children attending schools of this identity.*

**To:**
*The increased connectedness between Orthodox Protestant identity schools, combined with lower MMR coverage, may contribute to the formation of clusters of unvaccinated children, potentially increasing the risk of larger outbreaks of Measles, Mumps, and Rubella among children attending these schools.*

**Line 493:**
**From:**
*This provides a mechanistic explanation for the difference in outbreaks observed in the Orthodox Protestant and Anthroposophic communities in the past, where those in Anthroposophic communities have remained relatively small (a small number of schools), whereas the outbreak in 2013 (largely in the Orthodox Protestant community) was much larger.*

**To:**
*This offers an explanation for the differences in outbreak sizes observed between the Orthodox Protestant and Anthroposophic communities in the past, with outbreaks in Anthroposophic communities typically involving fewer schools, while the 2013*

*outbreak, which predominantly affected the Orthodox Protestant community, involved a larger number of cases*

2. At line 89-90, "This rise is driven by parental hesitancy towards vaccines and reduced MMR vaccination ... " I would rephrase to say “driven in part by” (otherwise too much of an absolute statement). Perhaps also include guardians but leave that op to you. If you can veer away from the word 'hesitancy' that would also be welcomed.

Thank you for this suggestion we have now incorporated it into the manuscript. The section now reads:
*This rise is partly driven by concerns of parents and guardians regarding routine childhood vaccines, in particular MMR vaccination rates reduced markedly during and after the COVID-19 pandemic.*

3. Line 91-92, "This increase in unprotected children raises fears of increased measles outbreak risk as has been documented in the past for measles and other highly infectious pathogens" - we suggest: The increase in unvaccinated children increases the risk of measles outbreak risk as has ..."

Thank you for this suggestion we have now incorporated it into the manuscript. The section now reads:
*This increase in unprotected children raises fears of increasedincreases measles outbreak risk as has been documented in the past for measles and other highly infectious pathogens*

4. Line 98-99 "Apart from hindering efforts to eliminate measles ... " please rephrase and use alternative phrasing for 'hindering'. In a way, I am not sure it is needed and find that "Measles outbreaks pose immediate risks to vulnerable populations, including young children who can't yet receive vaccinations and people with certain medical conditions." works quite well without the 'Apart from hindering efforts to eliminate measles ' part. I leave it up to your discretion.

Thank you for your suggestion. We have now removed the first part of the sentence.

5. Line 102 - 103, "The phenomenon of clustering of unvaccinated individuals within social groups is particularly clear in the Netherlands, where the ...". We like to suggest: ""The phenomenon of clustering of unvaccinated individuals within social groups is well documented in the Netherlands [add references here], where the ... [also needs a reference for the number you have included].

Thank you for your suggestion. We have now adapted the text to read:

*The phenomenon of clustering of unvaccinated individuals within social groups in the Netherlands is well documented, where the overall uptake has been high (>98% MMR1 by age 2) for nearly 30 years.*

6. Line 109, philosophies[16,17]. A space seems to be missing between 'philosophies' and '[16,17]'.

Thank you for pointing this out. We have addapted this.

7. This is a kind reminder that Supplementary materials

   Please note that supplementary materials are not checked and will be posted as supplied by the authors. Therefore, please double check. Please cite your Supporting Information as outlined here: https://journals.plos.org/plosmedicine/s/supporting-information - Please note you may use almost any description as the item name of your supporting information as long as it contains an "S" and number. For example, "S1 Appendix" and "S2 Appendix," "S1 Table" and "S2 Table. Please ensure each supplementary material has a call out (link) from your main manuscript.

Thank you for your reminder. We have now adapted the text to refer to "supplement S1 Text" consistently throughout.

----------------------------------------------------------

Comments from Reviewers:

Reviewer #1: The authors have substantially addressed my concerns and fixed the typos as suggested. The manuscript has been improved.

Please refer to the attached manuscript for my additional edits of typos.

Thank you kindly for your time and effort in improving our manuscript. We have incorporated all of your suggestions. The one exception was the suggestion to un-capitalise *Leigenalg*, which refers to the name of a python package and we believe it should remain capitalised.

Reviewer #2: I thank the authors for their careful consideration of all reviewers' comments and feel satisfied with their changes and clarifications. There are a few typos still floating about, but nothing that would significantly subtract from the paper's message.

Thanks again for your comments and suggestions that have strengthened our manuscript greatly. We have now adapted the manuscript another time to adress remaining typos.

Reviewer #3: The authors have revised their paper and this now represents an improved manuscript, acceptable for publication

Thanks again for your comments and suggestions. We are grateful for your time and effort in imporving our manuscript.
