# Supplementary material for: Estimating the risk and spatial spread of measles in populations with high MMR uptake: Using school-household networks to understand the 2013 to 2014 outbreak in the Netherlands
Source: PLoS Med. 2024 Oct 8;21(10):e1004466. doi: 10.1371/journal.pmed.1004466 (PMC11495615; doi:10.1371/journal.pmed.1004466)
Supplement: Attachment — Submitted filename: comments.docx [file pmed.1004466.s002.docx]

Thank you for the invitation to review the manuscript by Munday et al. Given school-specific MMR vaccine uptake, the authors created a national school-household network to quantify the outbreak risk of measles in the Netherlands. By simulating measles outbreaks on the network parameterized on 2013 data, the authors were able to explain the observed size and spatial distribution of 2013/14 outbreak, and identified that the highly connected orthodox Protestant schools associated with low vaccine uptake contribute to large outbreaks. The findings are important for understanding why measles outbreaks still occur in countries with high MMR vaccine uptake, and for archiving the WHO goals of eliminating measles.

However, I am concerned about whether it is adequate for reproducing the historical outbreak by comparing only the final size and spatial distribution without considering the epidemic curve. The authors acknowledge in the limitations that the current model cannot model temporal dynamics, but why can the results have important implications for understanding the timing of large measles outbreaks in the Netherlands (Lines 421-422)?

The authors think that their analysis can be repeated on an annual basis to assess the outbreak risk (Line 410). However, in the following discussion on implications, the authors also believe that the future outbreaks are likely to follow similar patterns among communities, if the population structure remains comparable in years to come (Lines 442-444). I am a bit confused whether it is necessary for the public health authorities to conduct the analysis annually.

Moreover, the manuscript is full of formatting typos, including capitalization, punctuation, abbreviations, references etc., which the authors should carefully proofread. Below are those typos to be fixed.

Line 37, change “Network” to “network”.

Lines 51-52, add the following reference to support the statement that many countries are facing challenges in Measles outbreaks.

<https://www.nature.com/articles/d41586-024-00265-8>

Lines 66-67, the sentence “each of these… were in children” is difficult to understand. Please improve it.

Line 112, change “school aged” to “school-aged”.

Line 139, change “Supplementary” to “supplementary”.

Line 147, there are two “the”.

Line 160, the parentheses for equations (1) and (2) are incomplete. Add a period “.” After “denomination”.

Lines 166-168, variable *i* should be italic.

Line 170, the citation “Donker et al” misses “.” and the year.

Line 175, no space between figure number and panel label.

Line 176, “I” should be lowercase and italic *i*; This also applies to “i + 1”.

Lines 192-193, change “supplementary information” to “supplementary material”.

Line 193, the citation “Munday et al.” misses the year.

Line 195, change “Susceptible” to “susceptible”, “Infected” to “infected”.

Line 197, the citation “Klinkenberg et al” misses “.” and the year.

Line 206, in equation (4) there is a variable *q* without definition.

Line 216, no space between figure number and panel label.

Line 231, change “Details” to “details”.

Line 237, *V* should be *V_j_*.

Line 242, “et. al” should be “et al.”.

Lines 254 and 503, “disease” should use the plural form “diseases”.

Line 272, add “that” after “risk”.

Lines 274, 350 and 694, use a comma delimiter for 1000. This also applies to 1241 and 1619 (Line 361).

Line 279, change “Alternative Model” to “alternative model”.

Line 297, there are two “of”.

Line 310-311, for Table 1 title, change “School” to “school”, “Region” to “Province”.

For Table 1 content, change “MEAN” to “Mean”.

Although the expanded form of MPP is provided in the List of abbreviations (Line 502), please provide the expansions of MPP and 95% CI in the main text. This also applies to the following CHI (Line 326), BH (Line 329) and IQR (Line 351).

Line 317, change “figure 4 A” to “Figure 4A”, “orthodox” to “Orthodox”.

Line 324, delete “than expected” since there is “than would be expected at random” at the end of this sentence.

Line 325, change “figure 4 B” to “Figure 4B”.

Line 327, change “Mainstream” to “mainstream”, “With” to “with”.

Line 336, change “The” to “the”.

Line 339, I cannot find the "scatter plots of network distance against geographic distance”. Please insert a reference to the figure.

Lines 345-347, how 5.18×10^-3^ and 4.81×10^-3^ are compared with 0.54 pairs^-1^km^-1^ and 3.83 pairs^-1^km^-1^ as they have different units and magnitudes?

Line 349, change “National” to “national”.

Line 356, change “Alternative” to “alternative”, and delete “,”.

Line 360, change “Alternative” to “alternative”.

Line 363, change “figure” to “Figure”.

Lines 371-372, change “School” to “school”, “Spatial” to “spatial”.

Line 379, change “a” to “an”.

Line 393, change “some” to “certain”.

Lines 399 and 456, change “school aged” to “school-aged”.

Lines 406-407, move “(4-17 years old )” to the first occurrence of “school-aged children” in Line 112.

Line 408, delete “-” from “outbreak-risks”.

Lines 431, 433, 434 and 485, change “anthroposophic” to “Anthroposophic”.

Line 438, change “this” to “which”, and add reference to support the “observations of the school network in England, UK”.

Line 455, move “the” before “5-9”.

Line 456, change “pre-school age” to “preschool-age”.

Lines 457 and 460, change “pre-school” to “preschool”.

Line 459, change “since” to “considering”.

Line 460, move “tend to be smaller” between “settings” and “,”.

Line 466, “risk” should use the plural form “risks”.

Line 479, change “school age” to “school-aged”.

Lines 482 and 513, change “this” to “which”.

Line 501, MMR is only the abbreviation of Measles, Mumps and Rubella.

Line 506, change “Weighted” to “weighted”.

Line 514, change “ministry” to “Ministry”.

Line 539-541, change “AJVH” to “AJvH”.

Line 539, delete “,” before “conceived”.

Line 671, change “,” to “.”.

Line 677, add “[Here labelled “Reformed”]” after “(Reformatorisch)”.

Increase the font size of texts in Figure 2 and Figure 4.

Line 682, change “,” to “.”, “the” to “The”.

Line 682, add “and” before “the rest”.

Line 685, change “in” to “In”; add “,” after “panel”.

In the left panel of Figure 4B, change the x-axis label “Coleman Index” to “Coleman Homophily Index”.

Line 688-689, delete “[Here labelled “Reformed”]”.

Line 695, change “the” to “The”; delete “.” after “uptake”.

Line 697, add “,” after “uptake” and after “specificity”.

Reference 8 has been published. Please update.

References 14 and 20 are duplicated.

References 19 and 21 are duplicated.

Supplementary Information

Page 1, change “leiden” to “Leiden”.

Variable C should be italic, *C*.

Add “as” after “expressed”.

Page 2, variable i should be italic, *i*.

Line 2, $k_{iC}^{\mathrm{in}}.$ should be $k_{iC}^{\mathrm{out}}$; 8 should be *i*; P should be *C*.

Line 5, change “Is” to “This is”.

Page 3, caption of Figure S1, change “+” to $\gamma$; add “.” after “respectively”.

Page 4, Line 1, the “school *j*” should be “school *i*”.

Line 7, change “we” to “We”.

Line 8, change the reference number 19 to 34 in main text. Also, double check the reference number 19 in the last line.

Line 11, variable i should be italic, *i*.

Line 16, i.e., the last line, change “of” to “for”.

In main text, a gaussian offspring distribution is assumed; however, here a Poisson distribution is assumed. Which one is correct?

Page 5, Line 1, variable i should be italic, *i*.

Line 9, change “A” to “a”.

In Table S1, change “Denomination” to “denomination”, “Mainstream protestant” to “Mainstream Protestant”.

Page 7, caption of Figure S2, change “black” to “Black”.

Page 8, Line 7, add space between “and” and “10”.

Page 9, the texts in Figure S4 are not clear. Please increase the resolution of this figure.

For the figure caption, change “estimates” to “Estimates”, “Orthodox” to “orthodox”; the citation “Klinkenberg et al.” misses the year.
